# The Ramus Intermedius: A Bridge to Survival in the Setting of Triple-Vessel Total Occlusion

**DOI:** 10.7759/cureus.61288

**Published:** 2024-05-29

**Authors:** Aleksan Khachatryan, Robert TD Chow, Mukta C Srivastava, Tufan Cinar, Joel Alejandro, Margarita Sargsyan, Mohammed Rifat Shaik, Vahagn Tamazyan, Reyaz U Haque, Hakob Harutyunyan

**Affiliations:** 1 Department of Internal Medicine, University of Maryland Medical Center, Midtown Campus, Baltimore, USA; 2 Department of Internal Medicine, University of Maryland School of Medicine, Baltimore, USA; 3 Department of Interventional Cardiology, University of Maryland Medical Center, Baltimore, USA; 4 Department of Cardiology, "Heratsi" Hospital Complex № 1, Yerevan, ARM; 5 Department of Internal Medicine, Maimonides Medical Center, New York, USA; 6 Department of Cardiology, University of Maryland Medical Center, Midtown Campus, Baltimore, USA

**Keywords:** va-ecmo, coronary artery disease, left heart catheterization, cardiogenic shock, st-elevation myocardial infarction (stemi), atherosclerosis, endothelial shear stress, trifurcation, cto: chronic total occlusion, ramus intermedius

## Abstract

Coronary artery disease continues to remain the leading cause of mortality worldwide. Coronary blood supply is provided through the right and left main coronary arteries. The left main coronary artery (LMCA) in turn gives rise to the left anterior descending (LAD) and left circumflex (LCX) arteries. In some cases, LMCA may trifurcate into the ramus intermedius (RI) in addition to the LAD and LCX arteries. Atherosclerotic plaque formation and rupture with subsequent clot formation and occlusion of coronary arteries are the underlying mechanisms of myocardial infarction. Though the clinical implications of the presence of ramus intermedius (RI) are controversial some data suggest that the RI is associated with an increased risk of atherosclerotic plaque formation in the LMCA and the proximal LAD. Conversely, it has been proposed that the RI provides an additional collateral source of blood supply to the myocardium and may potentially contribute to improved survival. Case reports tout the benefits of RI, specifically in the setting of multivessel coronary artery occlusions. Whether it increases the risk of atherosclerotic plaque formation or whether it is protective has yet to be determined. We present a case of a 58-year-old male who presented with acute coronary syndrome and cardiogenic shock due to total ostial occlusion of LAD. The patient had also chronic total occlusions of the right coronary artery and LCX but a patent RI, which was the only source of blood supply to the myocardium and practically determined the patient’s survival. Additionally, we performed a literature review to identify similar cases, to support RI's potentially protective role in enhancing survival.

## Introduction

Despite remarkable advancements in the management of coronary artery disease (CAD), it remains the predominant cause of mortality worldwide, including in the United States [1,2]. Atherosclerotic plaque formation and rupture within the coronary circulation are the underlying mechanisms of acute coronary events leading to fatal outcomes. The blood supply to the heart through the right and left main coronary arteries is essential for the maintenance of cardiac function. The left main coronary artery (LMCA), in turn, bifurcates into the left anterior descending (LAD) and left circumflex (LCX) arteries in the majority of cases. 

The ramus intermedius (RI) is considered a variant coronary artery that falls within the limits of normality [3]. In 15-30% of patients, the LMCA trifurcates into the RI, in addition to the LAD and LCX [4,5]. Functionally, the RI predominantly supplies the high anterior and anterolateral segments of the left ventricle (LV) [6]. In general, culprit coronary atherosclerotic lesions and ruptures tend to occur in the proximal segments of coronaries close to bifurcations; the proposed mechanism is that this location is the point of low endothelial shear stress (ESS) [7,8]. ESS is the tangential frictional force exerted on endothelial cells by blood flow within arteries. When ESS becomes pathological, it can predispose to the development of atherosclerosis. Consequently, the RI, as an additional flow divider, may potentially contribute to the generation of additional pathologic shear stress, atherosclerotic plaque formation, and damage to the coronary endothelium [9]. Consistent with this hypothesis, some data have emerged, indicating that the presence of RI is associated with an elevated risk of atherosclerotic plaque development within the LMCA and the proximal segment of the LAD [10-12].

However, conflicting reports suggest a protective role of the RI, especially in cases of multivessel CAD [13]. The postulated mechanism is that the RI serves as an additional collateral vessel, augmenting myocardial blood supply [14-16]. To support the potential of the RI in having a protective role in survival, we present an exceptional case of a 58-year-old male who presented with acute coronary syndrome and cardiogenic shock due to total ostial occlusion of the LAD. He also had chronic total occlusions (CTO) of the right main and LCX but a patent RI, which served as the only source of blood supply to the myocardium and resulted in the patient’s survival. Moreover, we performed a literature search in PubMed, Google Scholar, and all other sources of the English language published until December 2023 to detect all similar cases regarding RI's essential role in contributing to survival.

## Case presentation

A 58-year-old man, with a medical history of type 2 diabetes mellitus, hyperlipidemia, hypertension (HTN), and chronic kidney disease initially presented to an outside hospital with a one-day history of chest pain and vomiting. Upon evaluation, he was initially diagnosed with diabetic ketoacidosis, acute kidney injury (AKI), and non-ST-elevation myocardial infarction (NSTEMI).

**Figure 1:**
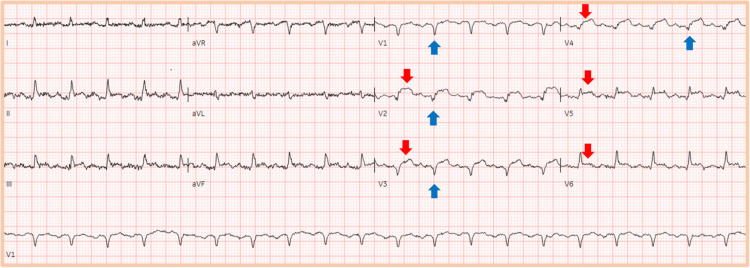
ECG Low voltage ECG demonstrating ST elevations in leads V2-V6 (red arrows) with Q waves in leads V1-V4 (blue arrows)

The chest pain was persistent, which was followed by the development of shock. Further evaluation revealed ST elevations in leads V2-V6 and Q waves in leads V1-V4 on electrocardiogram (ECG)(Figure 1).

He was diagnosed with ST-elevation myocardial infarction (STEMI), cardiogenic shock, and pulmonary edema. The management involved the initiation of epinephrine/milrinone, antithrombotic therapy, and the continuation of insulin. Additionally, an intra-aortic balloon pump (IABP) was inserted to support cardiac hemodynamics. Left heart catheterization (LHC) was deferred given a late presentation of STEMI (>36 hours), hemodynamic instability, and AKI. Subsequently, the patient was transferred to our cardiac intensive care unit for ongoing management of the cardiogenic shock.

There was no significant past cardiovascular history and his home medications included lisinopril, amlodipine, metformin, and glipizide. Family history was significant for the mother having had coronary artery bypass grafting surgery and the father having had HTN. The patient had stopped smoking more than 35 years prior, and he denied any alcohol consumption or illicit drug use.

The vital signs were as follows: temperature 37 °C, heart rate 109 beats per minute, blood pressure 108/69 mmHg on inotropic support, respiratory rate 18 breaths per minute, and oxygen saturation 92% on bilevel positive airway pressure (BiPAP). On physical examination, there was notable jugular venous distension (JVD). The cardiac auscultation demonstrated normal S1/S2, without murmurs, gallops, or friction rubs. Pulmonary auscultation demonstrated bilateral diffuse inspiratory crackles. The skin was cold and clammy. No peripheral edema was noted.

The initial laboratory testing, as shown in Table 1, was significant for increased troponin, NT-proBNP, leukocytosis, thrombocytopenia, transaminitis, and elevated creatinine.

**Table 1:** Initial laboratory results hs Troponin-I: high-sensitivity Troponin-I; NT-proBNP: N-terminal pro b-type natriuretic peptide; LDL: Low-Density Lipoprotein; Hemoglobin A1C: Glycated Hemoglobin; AST: Aspartate Aminotransferase; ALT: Alanine Aminotransferase

Laboratory parameter	Initial value	Reference range
hs Troponin-I	>3,600.0 ng/L	<35.0 ng/L
NT-proBNP	29,128 pg/mL	<900 pg/mL
LDL Calculated	43 mg/dL	0 - 99 mg/dL
Hemoglobin A1C	7.8 %	4.6 - 5.6 %
WBC	17 x 10*3/uL	3.1 - 9.5 x 10*3/µL
Platelets	67 x 10*3/uL	142 - 346 x 10*3/uL
AST	1,460 U/L	5 - 41 U/L
ALT	2,174 U/L	0 - 55 U/L
Creatinine	3 mg/dL	0.5 - 1.5 mg/dL

The initial transthoracic echocardiography (TTE), obtained while the patient was on inotropic support, revealed normal LV size with a left ventricular ejection fraction (LVEF) of 15%. The LV was nearly akinetic except for relative sparing of the basal-mid anterior and basal-mid anterolateral segments. The right ventricle was normal in size, with severely decreased systolic function (tricuspid annular plane systolic excursion (TAPSE)0.8 cm).

**Figure 2:**
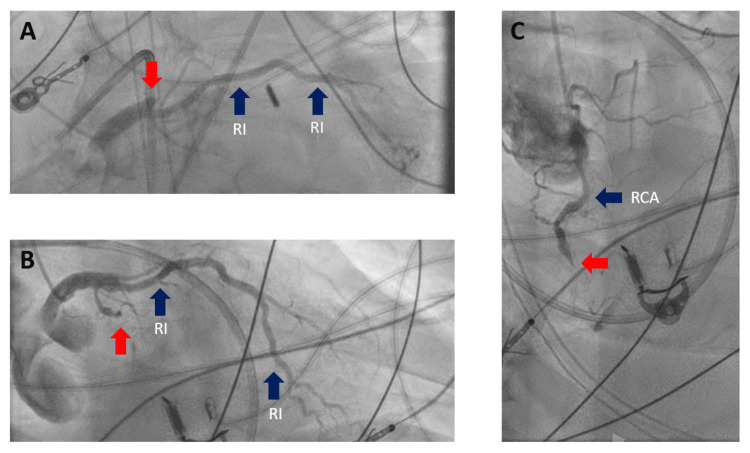
Left heart catheterization demonstrating 100% three-vessel occlusion Image A is the left anterior oblique caudal view, highlighting the occlusion at the ostium of the LAD artery. In image B, a right anterior oblique caudal view illustrates the proximal CTO of the LCX artery. Image C presents a right anterior oblique view showing the CTO of the mid-RCA. The occlusions are indicated by red arrows. RI: ramus intermedius; LAD: left anterior descending artery; RCA: right coronary artery; LCX: left circumflex artery; CTO: chronic total occlusion

The hospital course was complicated by progressive AKI, nonresponsive to diuresis, necessitating the initiation of continuous renal replacement therapy (CRRT). Concurrently, as a result of pulmonary edema, and deteriorating acute hypoxic respiratory failure attributed to cardiogenic shock, the patient was intubated and initiated on veno-arterial extracorporeal membrane oxygenation (VA-ECMO) on the second day following transfer. Eventually, the LHC on the fifth day after the initial chest pain revealed 100% ostial LAD occlusion, CTO of proximal LCX, and mid-right coronary artery (RCA) (Figure 2).

Importantly the patient had a moderate-sized patent RI that supplied collateral circulation both to the obtuse marginal (OM) branch of LCX and the RCA. Intervention on the coronary arteries was deemed inappropriate.

Following the initiation of VA-ECMO, the patient was extubated, the IABP was removed, inotropes/vasopressors were tapered off, and kidney function improved, leading to discontinuation of CRRT. Despite these advancements, persistent cardiogenic shock necessitated ongoing support with VA-ECMO, while the patient awaited heart transplantation. Ultimately, successful orthotopic heart transplantation (OHT) was performed 12 days following transfer. Pathological examination of the explanted heart revealed significant calcified coronary atherosclerosis, with evidence of a recent myocardial infarction involving the anterior LV wall and septum.

## Discussion

The patient’s STEMI presented in cardiogenic shock, necessitating inotropic and IABP support. The culprit lesion was likely the ostial occlusion of the LAD, based on the distribution of ST elevation on ECG and the pathologic confirmation of septal myocardial infarct. The TTE revealed a nearly akinetic LV except for the basal-mid anterior and anterolateral segments, which were supplied by RI. The viability of these segments, with mechanical and inotropic support, provided the necessary cardiac output crucial for survival and enabled transfer from a facility without ECMO capabilities with subsequent initiation of ECMO and OHT. The patent RI served a pivotal role while the patient was bridged on ECMO to OHT.

In our comprehensive review of the English literature, we could not identify any analogous cases with triple-vessel total occlusion in which the patient survived only due to the presence of patent RI. However, one report documented a similar scenario in which survival was attributed to the presence of both the patent RI and small (OM) arteries, despite having a triple-vessel total occlusion [13].

The RI is considered a variant coronary artery originating as a result of the trifurcation of LMCA, anatomically positioned between LAD and LCX. Rarely, in around 0.46% of cases, tetrafurcation of the LMCA is observed [17]. There currently exists no detailed criteria delineating the RI and distinguishing it from the high-origin diagonal or OM arteries. Thus, the exact prevalence of RI is inexact. One study found a significant discordance between coronary angiography (CAG) and intravascular ultrasonography (IVUS) in identifying RI. Specifically, 46% of CAG-based patients with RI were classified as having either high-origin OM or diagonal arteries upon IVUS interpretation [18]. One distinctive feature of RI is that it slides over the free surface of the LV wall rather than running through the anatomical groove, as in the case of the LAD and LCX arteries.

The presence of RI has advantages and disadvantages from a theoretical perspective. Specifically, a large-diameter RI can provide an additional collateral supply of blood to the LV anterolateral wall in cases of compromised coronary circulation. Conversely, the RI may have a diminutive effect on the diameters of the proximal LAD and LCX when located close to these arteries. However, if RI is precisely positioned between these arteries, its effect on their proximal diameter is minimal [19]. Additionally, some data indicate that the existence of RI is associated with an increased risk of branch point atherosclerosis [10,11,12,20]. These considerations underscore the complex interplay between RI anatomy and its potential implications for coronary artery health.

Atherosclerotic plaque formation tends to develop in locations of high curvature, points of bifurcation, or vascular branch point segments [21]. Increased bifurcation angle of the LMCA is also considered an independent risk factor for atherosclerotic plaque formation within the bifurcation region. It is proven that the laminar blood flow generates physiological ESS and is considered to have a protective role against atherogenesis [22]. However, anatomical branch points or bifurcations alter the laminar flow, which can result in the formation of pathological ESS and the generation of atherosclerosis [21]. The LMCA bifurcation region is a common site for altered laminar flow and is predisposed to atherosclerosis. Furthermore, the presence of RI in this region, as an additional flow divider, may further distort the laminar flow pattern and can potentially exacerbate atherogenesis. Moreover, the presence of RI increases the furcation angle, which in turn is considered an additional risk factor for atherosclerosis [23,24].

Some reports suggest that the presence of RI increases the risk of atherosclerotic plaque formation and obstructive lesions in the left main and proximal LAD [10,12]. Zhang et al. demonstrated that the presence of RI is not an independent risk factor for atherosclerotic plaque formation in the LMCA bifurcation zone, but it may indirectly increase the risk of atherosclerosis in the proximal segment of the LAD [11]. Another review highlighted the association between RI and larger anterior infarctions as well as more proximal LAD lesions [20]. Notably, this association was not observed in the case of the LCX, as the presence of RI was not associated with more proximal LCX lesions [25].

On the contrary, Abuchaim et al, suggested that the presence of RI may have a protective role against myocardial ischemia [14]. Moreover, there might exist a complementary relationship between diagonal, RI, and OM RI arteries; whenever one artery is absent or small in caliber, the other supplies its territory [15,16].

From a diagnostic perspective, occlusions of the RI may manifest as ECG changes involving leads I, aVL, or V5 and V6 [26]. However, it is also possible for RI occlusions to not be manifested on surface ECG, as lateral wall infarctions are not well reflected on surface ECG. Noninvasive or invasive diagnostic modalities as well as management options for RI lesions are not different from other coronary artery occlusions.

## Conclusions

In conclusion, the presence of an RI is associated with an increased risk of atherosclerotic plaque formation in the LMCA and proximal LAD. Conversely, some reports indicate a potential protective role of RI in improving survival. This case highlights the pivotal role of a patent RI in contributing to the survival of a patient with multivessel coronary artery occlusions, although there is no consensus regarding the clinical impact of the RI on cardiovascular mortality. Further research is essential to delineate the interplay between the RI and the overall cardiovascular outcomes of individuals with CAD.
